# Symbolic meanings of ordinary city streets and their trees

**DOI:** 10.3389/fpsyg.2022.1080025

**Published:** 2023-01-11

**Authors:** Bruce K. Ferguson

**Affiliations:** Retired, Pittsburgh, PA, United States

**Keywords:** interpretation, meaning, symbolism, streets, urban, trees

## Abstract

Symbolic meaning is one of a number of modes of humans’ relationships with physical settings. Although symbolic meaning is qualitative and ambiguous, it is an encompassing mode of interaction: symbolic meanings assemble feelings, urges, and abstract concepts; they shape people’s understanding of the world and motivate their purposes, attitudes, and actions. Early literature in environmental psychology acknowledged symbolic meaning’s promise, but in recent decades it has been inadequately studied; theoretical and methodological research has been needed. This paper advances the understanding and use of symbolic meaning by, first, presenting a theory which posits that in ordinary environmental settings symbolic meanings emerge from interaction between the perceptible qualities of environmental features and people’s psychological predisposition to respond to them. The paper then demonstrates methods which use the theory to objectively guide the identification of symbolic meanings in the case of ordinary urban streets and their trees. Although symbolic interpretation is intuitive and subjective, in this study it is guided by objective empirical knowledge and theoretical frameworks from human sciences. A combination of methods is applied, making conclusions answerable to diverse types of underlying data. One method was in firsthand observation of present-day streets; interpretations were accepted which linked objects’ perceptible qualities with people’s known dispositions to respond to them. A second method was interpretation in conventional street features’ documented historical evolution. Interpretations were accepted which linked objects’ perceptible qualities with people’s known disposition to respond, and with symbols’ known cultural tendency over time to specialize, differentiate, and evolve into coherent systems consistent with social norms. The results confirm that ordinary streets and their trees form a coherent system of symbols. Their meanings are social and sociomoral; they are guides to and affirmations of humane social life; they deserve to be prioritized in design agendas alongside tangible performance measures. It is concluded that symbolic meanings are present in ordinary urban settings, that their presence can be explained theoretically, and that their interpretation can be objectively guided.

## 1. Introduction

Symbolic meaning is one of a number of modes of humans’ relationships with physical settings (Proshansky, 1972). Symbolic meanings are important because they shape people’s understanding of the world and motivate their purposes, attitudes, and actions (Lakoff and Johnson, 1980, p. 3; Foster, 1994).

The analytical psychologist Jung (1964) and the art philosopher Coomaraswamy (1980) distinguished “symbols” and “signs” the same way. “Signs” are perceptible objects or images which denote other physical objects or actions, as does a crosswalk marking or a directional sign; their meanings are in the same physical realm with the objects. “Symbols” are objects or images which point to abstract concepts such as friendship or fairness; their meanings are in a different realm from that of physical objects. Symbolic meanings are broad, ambiguous, and often felt only unconsciously. Objects and images are important for conveying this type of meaning; words are comparatively limited in what they can express.

An environmental setting’s symbolic meanings differ from its concrete outcomes such as visual preference, psychological restoration, or healthy types of behavior. Such “instrumental” or “performance” outcomes are tangible and often capable of quantitative measurement (Stokols, 1990; Yang et al., 2015). Many of them are known from scientific study to be associated with desirable consequences such as economic efficiency or individual well-being. They are analyzed individually; their application logically dissects and divides the environment, and may inadvertently omit important aspects of the environment or of human life.

In contrast symbolic meanings assemble objects or images together with feelings, urges, and abstract concepts (Stevens, 1999, p. x). They are qualitative and often ambiguous. The meanings seen in given types of objects could vary with their cultural contexts and with the meanings of other nearby objects, with which they form interconnected symbolic systems. Broad symbolic meanings and discrete performance outcomes are complementary types of relationships between people and environment; they belong side by side in agendas for understanding and designing. A setting’s meanings are discovered not by discounting its numerous performance implications, but by adding alongside them a dimension of symbolic significance (Stevens, 1999, p. 7).

Cultural symbols in particular have meanings that are shared by the people in a society (Coomaraswamy, 1980; Rapaport, 1990). Anthropologists argue that a culture is a network of symbolic meanings; through them the culture is expressed and transmitted, and its social identity is shaped and sustained (Foster, 1994).

Symbolic meanings are discovered by interpretation, not by measurement. The interpretive act is necessarily intuitive and vulnerable to subjective bias (Geertz, 1973; Shankman, 1984; Keesing, 1987; Martin, 1993). Nevertheless investigating symbolic meanings, like any other scientific endeavor, is obligated to be based to the greatest possible degree on objective evidence. An interpretive study should seek out empirical knowledge at every possible step, and shape its conclusions consistently with it. Interpretation should demonstrate a fit between data and conclusions, preferably using multiple data sources and types (Seamon and Gill, 2016).

Early literature in environmental psychology acknowledged the presence and relevance of symbolic meaning (e.g., Proshansky, 1972). However in subsequent decades symbolic meaning (called ‘spiritual value’ or ‘metaphor’ in some literature) has been inadequately studied as a dimension of environmental psychology (Rapaport, 1990; Stokols, 1990; Tan, 2011). Links between physical form and symbolic meanings have not been well understood. Techniques for identifying and assessing symbolic meanings need to be developed.

This paper advances the understanding and use of symbolic meanings in environmental psychology by, first, presenting a theoretical model for explaining the origin of symbolic meanings in ordinary environmental features. It then uses the model to demonstrate methods for identifying meanings in the case of Western cities’ conventional residential city streets and their trees.

Ordinary residential streets are important because they comprise a large portion of cities’ overall street inventories and house correspondingly large proportions of city residents. They do not have the special, unique meanings which other city settings acquire from their religious, political, or historical associations. Nor do their trees necessarily have the separate meanings which some plantings acquire from their memorial purposes (Woudstra and Allen, 2022). If ordinary streets have symbolic meanings, they come through the perceptions of people who live their daily lives in them (Stokols, 1990).

The particular symbolic meanings of ordinary streets and their trees have been little investigated. Urban designers writing about streets (e.g., Anderson, 1991; Southworth and Ben-Joseph, 2003; Loukaitou-Sideris and Ehrenfeucht, 2011; Mehta, 2013) have focused on streets’ diverse physical functions, not symbolic meanings. Among psychologists Schroeder et al. (2006) pointed out that street trees have important meanings for people, but did not try to systematize them. Other psychologists have correlated urban trees with specific performance outcomes such as human health or social interaction (e.g., Coley et al., 1997; Wolf et al., 2020), or pointed out the general roles of trees in the human psyche (e.g., Schroeder, 1992; Dwyer et al., 1994; Sommer, 2003). Historians of street trees (e.g. Lawrence, 2006; Johnston, 2017; Dümpelmann, 2019) have noted social and political roles for trees, but not sought a psychological theory for street trees’ symbolic meanings.

Ordinary residential streets are convenient for interpretive research because their conventional arrangement of infrastructure and trees has been stable for two centuries and is now available for observation across a large part of the Western world. Their usual components of sidewalk, curb, vehicular pavement (“cartway”), and trees comprise a single behavioral and experiential setting. They are uncomplicated by civic and commercial digressions like street vending, sidewalk café service, taxi stands, or security barriers.

## 2. Theory: The origin of ordinary environmental symbolism

To explain the presence of symbolic meanings in ordinary environments and to guide their interpretation, this study adapts a theory of symbolic origin developed originally by analytical psychologists to help understand and manage people’s internal experiences.

In people’s internal experiences, memories, and imaginings generate images which have various types of visual qualities such as above-below, active-still, open-enclosed, and orderly-chaotic. Qualities like these have practical importance to people; they contain information relevant to people’s personal and social situations and choices of potential actions. So the images are symbols: upon them the mind, usually unconsciously, assembles feelings and motivations (Stevens, 1999; Kosslyn et al., 2001; Goodwyn, 2012; Colman, 2020). If people were to try to express their meanings verbally, they would have to use abstract concepts such as hope or fairness. Images communicate such abstract concepts more immediately and completely than could conscious verbal expression.

This study posits that the various kinds of visual qualities that occur in internally generated images occur also in externally perceived images, and that they assemble the same types of feelings and motivations: they are symbolic. Seeing an image with the eyes triggers the same brain regions as imagining it (Kosslyn et al., 2001). The defining difference between the original psychological theory and this environmental adaptation is the origin of the images: internal generation or external perception. The symbolic meaning is co-produced by the qualities of the environmental setting and the people who encounter them. It emerges from the interaction between them, to which the environmental setting brings its perceptible qualities, and the people bring their psychological predispositions to respond (Schroeder, 1992; Jelić et al., 2016).

Objects and images with symbolically relevant qualities are what Coomaraswamy (1980) called ‘natural’ symbols. Their meanings do not arise from conciliar agreement or arbitrary custom; they are latent in the images’ relevant properties in the context of their setting. New symbols could come into existence any time (Stevens, 1999, p. ix); anything in people’s lives could attain symbolic significance (Jaffé, 1964, p. 257). Lakoff and Johnson (1980), Stevens (1999), Tressider (2005), and Goodwyn (2012), reviewed many of these types of qualities and images; further sources for specific qualities will be cited in the Results section.

People always and automatically scan the environment for relevant qualities like these; they recognize their practical importance and feel their emotional import intuitively and immediately (Zajoric, 1980; Schroeder, 1992; Mace, 2015, p. xxii). They have begun to act in accord with them before they could express rationally what they have seen. Meanings rise to conscious awareness only in certain instances and circumstances.

The ordinary streets studied in this research are cultural objects; they are produced and adopted by a city’s people and institutions. Anthropologists argue that in cultural objects symbolic meanings are inevitable and ubiquitous, whether or not the people who produce and use them are consciously aware of their symbolic role (Rowntree and Conkey, 1980; Foster, 1994). The environments that societies build for themselves are symbolic expressions of their understanding of the world and the relations of its members to it and to each other (Firth, 1973, pp. 403–404). A culture as a whole is formed of the meanings that lie within its interrelated symbols.

Over time a culture tends to evolve with its appreciation and utilization of its symbols (Foster, 1994). Its symbolic objects tend to specialize, to differentiate, and to grow together into coherent symbolic systems which are consistent with social norms (Foster, 1994; Spillman, 2002). Surrounding people with recognizable symbols of social and moral norms, consistent with natural human dispositions, makes those norms and values seem ordinary, common, and shared (Harris and Lipman, 1980; Varnum and Grossmann, 2017).

## 3. Materials and methods

The subject of symbolic meaning requires qualitative research methods; this reality is well known in the fields of, for example, analytical psychology and cultural anthropology (Paul, 1987; Ashworth, 2008; Seamon and Gill, 2016; Yardley, 2017). The interpretation of symbolic meaning is an intuitive act; it is potentially vulnerable to subjective bias (Stevens, 1999, p. 8). With that in mind, in this study the scope of interpretation was limited by concentrating on only the few specific types of features that characterize ordinary residential streets. The researcher was already very familiar with the objects’ physical parameters and functional relationships from previous experience in urban design. The theory of symbolic meanings’ origin described in the previous section was used to objectively guide the features’ interpretations by linking perceptible qualities with people’s objectively known psychological dispositions to respond to them. Specific relevant types of links and research that supports them will be cited at appropriate points in the Results section.

The interpretation was conducted in two different methods, making the conclusions answerable to two different types of data (Seamon and Gill, 2016). One method was in firsthand observation of present-day streets; the second was in the documented history of features’ physical evolution. The following sub-sections describe each of the two specific methods.

### 3.1. Interpretation of present-day streets

Symbolic meanings in present-day streets were interpreted in firsthand observation. For that purpose a sample of existing present-day streets was constructed. Initially, approximately 200 residential streets in North America and overseas were preliminarily reviewed from the author’s previous national and international travels, internet street views, and general knowledge of urban form and history. From them approximately 20 were selected for detailed and repeated observation. In that sample an effort was made to include streets with representatively diverse histories, social conditions, and design details. Ancient streets which had never experienced modern design evolution were excluded.

Detailed observations were conducted during 2020 and 2021 using personal visits, photographs collected during prior travels, and online street views. Some were observed iteratively as alternative hypotheses were considered. Perceptible qualities were noted. The qualities were interpreted by searching scientific and philosophical literature for their potential symbolic meanings. Interpretations were accepted which (1) linked perceptible qualities with known human dispositions to respond, (2) could not be equally well explained by physical function alone, and (3) were consistent with interpretations found in historic evolution, which was being reviewed at the same time the firsthand observations were being made.

**Figure 1:**
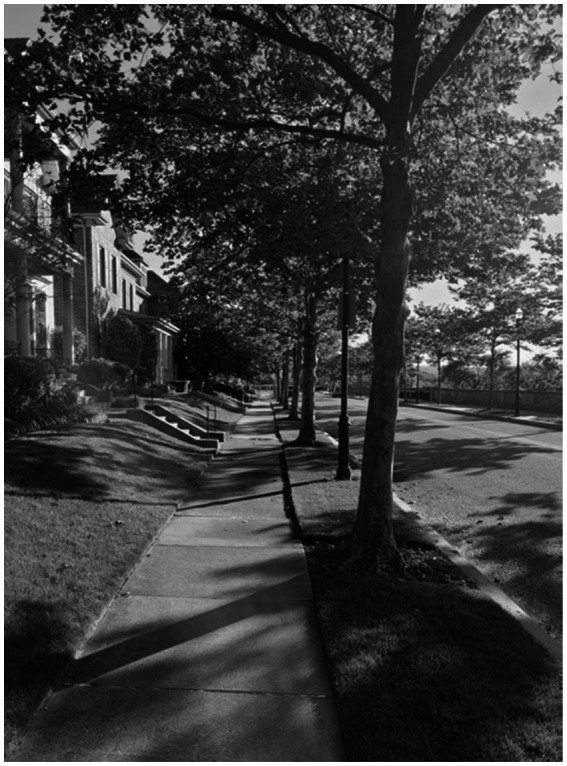
One of several repeatedly observed streets in the Summerset neighborhood of Pittsburgh, Pennsylvania.

Particularly numerous repeated observations were made in the Summerset neighborhood of Pittsburgh, Pennsylvania. Its streets are well supplied with orderly street infrastructure and numerous street trees, producing the full symbolic potential of today’s conventional streets. A typical example is shown in Figure 1. Hundreds of daily walks were taken there in all seasons, weathers, hours of day and night, and types of neighborly interactions.

### 3.2. Interpretation in cultural evolution

Symbolic developments in streets’ historic evolution were interpreted from review of published historical literature. Incremental steps in physical development were identified: at each step some feature or perceptible quality was added, or replaced an older feature or quality. New features’ perceptible qualities and potentially symbolic implications were noted. Interpretations were accepted which (1) linked perceptible qualities with known human dispositions to respond, (2) could not be adequately explained by physical function alone, and (3) showed progressive physical and symbolic differentiation, specialization, and assembly into coherent systems.

In both types of interpretative methods, five alternative hypotheses of symbolic meaning were entertained:

1. Spatial order: Human beings need orientation for navigating and finding their way physically and functionally (Gombrich, 1984, pp. 2–5). People actively (usually unconsciously) scan the environment, searching for perceptible patterns that they can understand and use.
2. Social order: A setting’s pattern of behavioral norms is its social order. Behavioral norms are a basis for people to predict each other’s behavior, to maintain communication with each other, and to shape their relationships and identities in the local community (Cialdini and Goldstein, 2004; Carrus et al., 2009; Lawler et al., 2015). Social interactions can be governed by social norms in settings that are socially ordered and defined. Stable patterns of behavior can emerge from interactions between people where they are not disrupted by non-social features or events in the environment (Lawler et al., 2015). A defined place, affording human presence and protected from non-social disruptions and distractions, can allow focused, sustained interactions and the emergence of order from them.
3. Psychological restoration from urban stress: Natural environments are associated with psychological restoration of mood and focus as a relief from urban stress (Kaplan and Kaplan, 1989; Hartig and Staats, 2003). People who encounter street trees in the midst of cities might feel that the trees are representatives of natural landscapes, symbolizing environmental conditions and mental states conducive to well-being.
4. World tree (sometimes called “cosmic tree” or “tree of life”): In ancient cultures, a tree symbolically unites or shows the relationship between heaven and earth; it is a symbol of principles underlying the whole of creation (Tressider, 2005, pp. 484–485). If street trees have this meaning, they would bring a concept of cosmic order into quotidian lives.
5. No significant symbolic meaning: If street forms can be adequately explained by physical functions such as drainage or safety, without respect to any symbolic meaning, then the entire concept of symbolic meaning for city streets could be discarded.

## 4. Results

The results are presented here first as a narrative of streets’ historical development, with reference to symbolic implications. The symbolic experience of present-day streets will be presented second.

### 4.1. History of physical and symbolic evolution

Modern street forms evolved out of the conditions of medieval streets, which were physically unarticulated except for a central gutter. In that setting pedestrians mingled with horse-drawn vehicles and occasional mixtures of dogs, pigs, and poultry (Corporation of London, 2005; Jørgensen, 2008). Typical human beings must have considered those conditions chaotic and unattractive for any social purpose. Municipal councils monitored the conditions, counting on fronting homeowners to share responsibility for street maintenance.

Change began in the decades after London’s Great Fire of 1,666, when many London districts were redeveloped, or were developed for the first time to accommodate rapid growth. The metropolis saw itself as an emerging world capital, and its various districts used street improvements, among other things, to compete for growth and prestige (White, 2010). Under a series of city ordinances, pedestrian sidewalks were distinguished from cartways with smooth pavements and bollard outlines. The cartways retained their rough pavements and central gutters (Corporation of London, 2005, pp. 6–7, Lawrence, 2006, p. 177). This distinction functionally specialized and protected the pedestrian way, where social interactions could be humanely governed by behavioral norms. The functionally differentiated streetscape was a symbol of social order. It marked a clear border between the city’s social order and what was disorderly and impure. Explicitly displaying the definition of what is culturally contemptible and excluded defines and orders a society (Douglas, 1966, pp. 2–3). To reject contaminating forces is a social virtue (Horberg et al., 2009).

In the late 18th century, a moral dimension was added to the distinction. Residents of European cities were becoming less tolerant of obnoxious odors and more conscious of connections between cleanliness and health, while civic authorities were growing more active in managing city life (Jørgensen, 2008). Under a new series of London acts, sidewalks were raised perceptibly above the cartway by curbs, with gutters at the bottom. The curbs symbolically elevated the sidewalks’ humane social life above the cartway. People naturally sense physical ascent as elevation in virtue and status (Meier and Robinson, 2004; Meier et al., 2007; Ścigała and Indurkhya, 2016; Zhai et al., 2018). When we lift upward we focus and exert; we control our lives and enact our purposefulness; when we fall downward we are failing (Bevan, 1962, pp. 25–26; Schubert, 2005; Cian, 2017). The cartways remained disorderly and impure (Corporation of London, 2005, p. 7, White, 2010). The damp gutter below the curb was a warning of taboo beyond. We speak of gutter talk, mind in the gutter; twentieth-century Nazis made Jews walk in the gutter. The new curb-and-gutter arrangement was quickly adopted in other European cities (Corporation of London, 2005; Lawrence, 2006; White, 2010).

As for trees, socially relevant tree symbolism was discovered in the 17th and 18th centuries, in elite garden-like reserves at the edges of Paris and other cities, away from the old medieval streets. Trees were aligned with the gardens’ walkways for elite promenading (Lawrence, 1988). Trees are living things with which people naturally feel likeness. Their ascending growth symbolically elevated the gardens’ elite society in virtue and status (Dwyer et al., 1994; Bloch, 1998; Rival, 1998; Ścigała and Indurkhya, 2016).

In the early 19th century, Parisian officials introduced trees to functional city streets while upgrading the city center’s old medieval streets. They joined recently developed infrastructure from Britain with trees from French gardens. The trees were placed on the level of the sidewalk, in a limen separating the sidewalk from curb, gutter, and cartway. By aligning with functional street infrastructure the trees participated in and reinforced the streets’ symbolic social order. By ascending overhead they extolled the virtue of the sidewalk’s pure social life (Lawrence, 1988; Lawrence, 2006, pp. 190–193; Johnston, 2017). This was a symbolic synthesis which drew immediate international attention, especially during Haussmann’s large-scale redevelopment of central Paris slightly later in the 19th century (Lawrence, 1988, 2006). Many European cities adopted the model in their central boulevards and elite neighborhoods. It then dispersed into wider residential districts as middle-class populations grew, manifesting natural human concern with the quality of domestic life. From the mid-nineteenth century it became routine in new and redeveloped urban streets in large parts of Europe, North America, and the rest of the Western cultural world (Lawrence, 2006, pp. 193, 199, 208, and 219).

In the generations since, the Parisian model has endured two tests of its symbolic role, as the physical character of cartway traffic changed. The first was based on 19th century horse traffic, which increased as cities’ populations and industries grew and living standards rose. People and freight were being carried in great numbers of horse-drawn wagons and carriages. Abundant horse manure made cartways offensively filthy and smelly (Tarr, 1971; Winter, 1993, p. 13; Corporation of London, 2005, p. 10; Morris, 2007).

The second test came at the beginning of the twentieth century, when automobiles abruptly replaced horses. This ended the manure, but the autos brought a new kind of inhumanity to the cartway. The cars’ drivers were enclosed, hidden, anonymous, and speedy. Cartway interaction was asocial and inhumane (Clow, 2004; Dey and Terken, 2016; Rasouli et al., 2017; AlAdawy et al., 2019). Ordinary people were repelled by the cartway’s new inhuman interaction as they had been by its previous pollution. Within a couple of decades cartway behavior was moderated with lane markings, stop signs, and jaywalking rules, but its anonymity and a sociality remained (Norton, 2007). For the sidewalk, rules evolved that protected public pedestrian use (Loukaitou-Sideris and Ehrenfeucht, 2011).

Through both tests the Parisian symbolic synthesis persisted. The cartway retained the symbolic form of that moral category; whatever was in the cartway was repulsive in the feelings of ordinary people, and was shunned (Haidt et al., 1997). Humans remained above it amid their affirming and extolling symbols. The Parisian model’s stability over time confirmed that it represented important psychological and cultural meanings (Winter, 1993; Morris, 2007; Nixon, 2014). It continued to be adopted and dispersed in growing cities throughout the Western world (Clow, 2004, Corporation of London, 2005, p. 10).

### 4.2. Meanings in present-day streets

The symbolic meanings found in firsthand experience of today’s streets were consistent with those inferred from their historic evolution. Physically, their conventional arrangement is simple, consistent, and easily understood. Functionally, the sidewalk and cartway afford and guide human actions with smooth, continuous support and clear, continuous edges.

People tend to follow streets’ functional guides and behavioral norms. Social interaction among the sidewalk’s people is direct, intuitive, and humanely personal, combining speech, facial expressions, and manual and bodily gestures (Jacobs, 1961; De Dtefani and De Marco, 2019). Residential neighbors greet and part, acknowledging their identities and social relationships (Firth, 1973, pp. 299, 404). Even strangers interact with movements and glances. These almost unconscious types of communication share human intentions and feelings while negotiating positions and actions in the shared space.

On the cartway, interactions between pedestrians and drivers, when they occur, tend to be nonpersonal and nonhuman. Autos’ enclosure hides drivers’ faces and gestures. They are disembodied, without human identity, and alienated from the social environment (Nixon, 2014). Normal intuitive paths of human communication such as eye contact, facial expression, and subtle gestures are blocked. Pedestrians do not know what drivers’ attitudes or intentions are.

Street trees are aligned with the infrastructure and its functions. Consequently they participate in and reinforce the streets’ symbolic social order. In addition they influence residents’ images of their community and their places in it (Sommer, 2003). They are affirmations of the good that is in street life, now that symbolic forms have evolved to define and order it. The personal feelings that people today express about street trees tend to be abstract and personal such as “sense of humanity and family”, “sense of community”, and “spiritual values” (Schroeder et al., 2006). Where municipalities have attempted to retrofit trees into streets that had not been designed to hold them, residents have complained; those streets lacked a limen ready for planting, so planting required cutting away sidewalk space, injuring residents’ sense of territoriality over the sidewalk space (Rae et al., 2010).

## 5. Discussion

**Figure 2:**
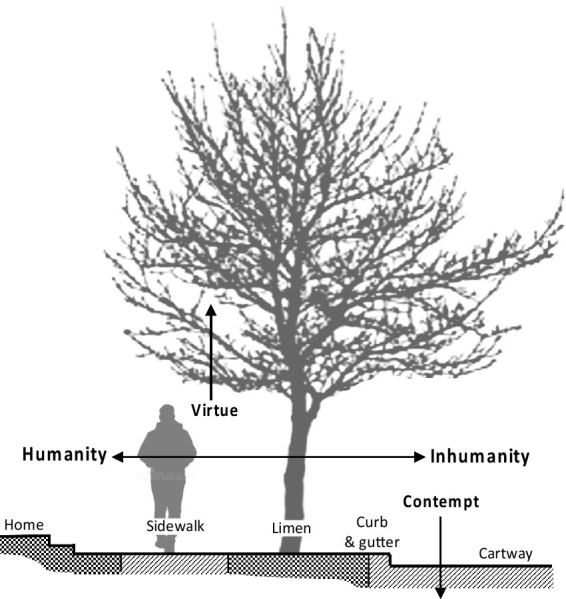
Summary of symbolic meanings in today’s conventional streets and street trees.

The study’s results indicate that today’s conventional model of ordinary streets and their trees is a coherent system of symbols. Its meanings are social definition, order, and purity. It morally ranks different modes of social interaction. Like other systems of cultural symbols known to anthropologists, it embodies and communicates the way its social life is lived (Douglas, 1966, p. 3). That today’s conventional model of ordinary residential streets is widespread and stable indicates that it manifests meanings fundamental to human nature and society. Figure 2 summarizes these meanings. On the left the humanity of personal, intuitive social interaction is enacted on the sidewalk. On the right the inhumanity of anonymous, alienated interaction is enacted in the cartway. Between them the curb and the gutter physically separate and morally rank them. The limen further separates them; from it trees ascend further above to extol the virtue of the pure social realm.

Of the five hypotheses of symbolic meaning entertained in this study, these results confirm those of spatial and social order, although the moral aspect of social order, introduced to streets by the curb and gutter and then by symbolically ascending trees, had not been anticipated.

The hypothesis of trees’ nature-restoration effect was rejected. Street trees’ symbolic meanings, like those of other symbolic objects, are conditioned by those of other nearby objects. Although trees are living things derived from nature outside human society, their restorative interpretation cannot be transferred into the different context of city streets without appropriate modification. In the context of city streets trees are intimately surrounded by and aligned with social artifacts, functions, and purposes. Their symbolic role has evolved together with street infrastructure, in the manner expected for cultural symbols by anthropologists (Foster, 1994; Spillman, 2002), into a single coherent symbolic system in which street trees participate in and reinforce the city’s social norms and purposes, not divert attention from them. A qualification of this conclusion is that in city streets trees might have a secondary meaning of the kind of restorative effect that they have in nature, hidden beneath their dominant social meaning. If it is present, it would be hard to separate from trees’ affirmative social symbolism.

The hypothesis of trees’ cosmic-order symbolism was similarly rejected in favor of their social role and with a similar reservation that some secondary meaning, not specifically social, may lie beneath the dominant social meaning.

The hypothesis that conventional streets and their trees have no symbolic meaning was rejected because a symbolic role was clearly present at every stage in the evolution of their forms, while physical functions played comparatively little role. Distinguishing sidewalk from cartway with new pavement and bollards did not separate people from physical danger; it distinguished the pure social order. Adding curbs while removing bollards was not a net improvement in safety. Moving gutters from the center of cartways to the sides did not make drainage more efficient. Adding trees did not improve any physical function. Changes in the cartway’s traffic types, in which human psychological response remained categorically constant while physical hazards changed, were not followed by alterations of street form.

Within the pattern of components referenced in Figure 2, individual streets were found to differ in physical and symbolic details in accord with all sorts of economic, demographic, cultural, and historical circumstances. Different details reflect and shape residents’ ideas of their communities and their places in them. Streets that clearly symbolize social order locate, guide, and encourage residents in a communal social landscape; ambivalent designs express correspondingly weak levels of social identity (Rapaport, 1990, pp. 137, 191; Lawrence, 2006, p. 227; Von Bergsdorff, 2007).

Table 1 lists examples of streets with three different levels of symbolism of social order, and the various detailed features that shape them. Streets strongly symbolizing social order have all the features illustrated in Figure 2, without disorderly intrusions such as overhead wires. Other streets’ social symbolism is relatively weakened by ambiguously sloping ‘wedge’ curbs, scattered or irregular trees, or overhead wires (often misaligned with street infrastructure and each other). Weak symbolism was found in old poor neighborhoods where maintenance of infrastructure and vegetation has been negligent. Equally weak symbolism was found in wealthy suburbs where meanings stress domestic privacy or immersion in local natural environments over symbols of shared social order.

**Table 1:** Features of selected streets with different levels of symbolic social order.

Relative strength of symbolism of social order	Example	Infrastructure features	Tree features	Residential character	Other context
Strong	Grace Street, Church Hill, Richmond, Va.	Vertical curb, two sidewalks, brick-paved limens	Numerous, regularly spaced street trees, no overhead wires	Single-family homes, attached homes, and apartments, middle to upper middle income	Installed 1880±
Moderate	Whipple Street, Swissvale, Pa.	Vertical curb (obscured by pavement overlays), two sidewalks, grass limens	Scattered street trees, some overhead wires	Single-family homes, lower-middle income	Installed 1900±
Moderate	Liberty Blvd., Traditions of America, Canonsburg, Pa.	Wedge curb, one sidewalk	Regular street trees, no overhead wires	Single-family homes, middle and upper-middle income	Installed 2020±
Weak	Ridgedale Lane, Fox Chapel, Pa.	Wedge curb, no sidewalk	No street trees, no overhead wires	Single-family homes on large lots, upper income	Installed 1960s
Weak	Apple Street, West Homewood, Pittsburgh, Pa.	Vertical curb (obscured by pavement overlays), one sidewalk, no limen	No street trees; overhead wires; overgrown vegetation	Single-family homes, low income	Installed 1900s
Weak	Antler Court and Deer Crossing, Cardinal Ridge, Medford, NJ	No curb, no sidewalk, no lawns	No planted trees (dense woodland setting), no overhead wires	Single-family homes on small clustered lots, middle-to upper-middle income	Installed 1975±
Weak	Eagle Ridge Road NE, Albuquerque NM	Discontinuous curb, no sidewalk	No planted trees (desert setting); no overhead wires	Single-family homes on large lots, upper income	Installed 1990±

## 6. Conclusion

This study’s findings confirm that symbolic meanings are present in today’s ordinary urban settings, where they act as one of the dimensions of relationship between person and environment. Although the feelings, urges, and abstract concepts assembled in a symbolic setting are qualitative and ambiguous, symbolic meaning is a more encompassing dimension of interaction with environment than quantitative performance measures. The meanings of ordinary streets found in this study are guides to and affirmations of human social life; they deserve to be prioritized in design agendas alongside tangible performance measures.

This outcome supports the theory of symbolic meanings’ origin in the interaction between the perceptible qualities of the environment and the psychological disposition of people to respond. Meanings are shaped in detail by varying social, cultural, and historic circumstances.

Although the act of interpreting symbolic meanings is qualitative and subjective, it can be objectively guided by empirical knowledge and theoretical frameworks from the human sciences. Combining observations of present-day features with review of historic evolution diversifies data sources and multiplies cases that are observed. Interpretations of meaning can be accepted which link objects’ perceptible qualities with human psychological disposition to respond, and have evolved over time into physical and symbolic differentiation, specialization, and assembly into coherent systems.

## Data availability statement

The original contributions presented in the study are included in the article/supplementary material, further inquiries can be directed to the corresponding author.

## Author contributions

BF conceived the study, collected all data, made the concluding interpretations, and wrote all sections of the paper.

## Conflict of interest

The author declares that the research was conducted in the absence of any commercial or financial relationships that could be construed as a potential conflict of interest.

## Publisher’s note

All claims expressed in this article are solely those of the authors and do not necessarily represent those of their affiliated organizations, or those of the publisher, the editors and the reviewers. Any product that may be evaluated in this article, or claim that may be made by its manufacturer, is not guaranteed or endorsed by the publisher.
